# Colocalised Genetic Associations Reveal Alternative Splicing Variants as Candidate Causal Links for Breast Cancer Risk in 10 Loci

**DOI:** 10.3390/cancers16173020

**Published:** 2024-08-29

**Authors:** André Besouro-Duarte, Beatriz Carrasqueiro, Sofia Sousa, Joana M. Xavier, Ana-Teresa Maia

**Affiliations:** 1 CINTESIS@RISE, Universidade do Algarve, 8005-139 Faro, Portugal; a65440@ualg.pt (A.B.-D.); jgxavier@ualg.pt (J.M.X.); 2 Faculty of Medicine and Biomedical Sciences, Gambelas Campus, Universidade do Algarve, 8005-139 Faro, Portugal; 3 Centro de Ciências do Mar (CCMAR), Universidade do Algarve, 8005-139 Faro, Portugal

**Keywords:** breast cancer risk, genome-wide association studies, alternative splicing, splice quantitative trait loci, colocalisation analysis

## Abstract

**Simple Summary:**

Hundreds of common genetic variants have been linked to breast cancer, but their exact mechanisms of action remain unclear. Understanding these mechanisms could lead to better prevention strategies and improved survival rates. Our study focused on how these variants influence splicing—a process by which a gene’s coding elements are rearranged to produce different proteins. By analysing data from healthy breast tissue, we identified 43 variants within twelve genes associated with both alternative splicing and breast cancer risk. We then used advanced computational tools and existing experimental data to explore the biological significance of these findings.

**Abstract:**

Genome-wide association studies (GWASs) have revealed numerous loci associated with breast cancer risk, yet the precise causal variants, their impact on molecular mechanisms, and the affected genes often remain elusive. We hypothesised that specific variants exert their influence by affecting cis-regulatory alternative splice elements. An analysis of splicing quantitative trait loci (sQTL) in healthy breast tissue from female individuals identified multiple variants linked to alterations in splicing ratios. Through colocalisation analysis, we pinpointed 43 variants within twelve genes that serve as candidate causal links between sQTL and GWAS findings. In silico splice analysis highlighted a potential mechanism for three genes—*FDPS*, *SGCE*, and *MRPL11*—where variants in proximity to or on the splice site modulate usage, resulting in alternative splice transcripts. Further in vitro/vivo studies are imperative to fully understand how these identified changes contribute to breast oncogenesis. Moreover, investigating their potential as biomarkers for breast cancer risk could enhance screening strategies and early detection methods for breast cancer.

## 1. Introduction

Genome-wide association studies (GWASs) have pinpointed numerous variants linked to alterations in breast cancer risk, predominantly situated in non-coding regions of the genome [1,2]. Some of these variants have been identified as regulators of gene expression mechanisms through SNPs in enhancer/promoter regions or miRNA that modulate transcript stability and translation [3,4]. Nevertheless, the specific mechanisms underlying most of these associations remain unclear.

Alternative splicing (AS) is a process that enables a single gene to generate multiple proteins by splicing in/out functional domains. Approximately 70% of human genes exhibit alternative splice forms, expanding the repertoire of functional transcripts and proteins by adding/removing functional blocks [5,6]. Variations in splicing regulatory elements can modify motif recognition by RNA-binding proteins, leading to differences in isoform ratios or even the creation/destruction of motifs, generating new isoforms through cryptic splicing or mis-splicing [7,8]. Furthermore, splicing is specific to tissue/cell types and developmental stages, resulting in distinct phenotypes that necessitate independent analysis [9].

Previous research demonstrated the influence of AS on various diseases, including cancer [8], where splicing changes can act as oncogenic drivers [10,11,12]. In cases with *BRCA1* germline mutations, cis-regulating mutations modulate AS, leading to its inactivation—a feature often replicated in tumours [13,14]. Another instance is *ESR1*, which encodes for the oestrogen receptor; alterations in the ratios of alternatively spliced isoforms are associated with an elevated cancer risk [15]. Another two studies attempted to establish more systematic associations between GWAS variants and alternative splicing but did not evaluate AS in the normal tissue, the most relevant for risk studies, and carried out sQTL in a small number of variants [16,17].

This paper proposes to comprehensively assess the impact of alternative splicing cis-regulatory variants on breast cancer risk by leveraging previously collected genomic data and employing robust analysis methods. Specifically, we aim to quantify alternative splicing in healthy breast tissue, identify variants associated with this mechanism, colocalise these variants with previously published GWAS findings on breast cancer risk to identify common variants, and conduct in silico analyses to propose candidate causal variants and elucidate the impacted molecular mechanisms.

## 2. Materials and Methods 

### 2.1. Data Sources

RNA-seq SRA and WGS-based VCF files were obtained from the GTEx v8.p2 study (dbGAP Study accession phs000424.GTEx.v8.p2). Anonymised sample attributes, phenotypes, and transcript TPMs were downloaded from the GTEx Portal. Data access was authorised on 7 March 2018, with retrieval in April 2018.

GWAS data for breast carcinoma traits were sourced in June 2020 using the gwasrapidd R package, leveraging the NHGRI-EBI GWAS Catalog’s REST API [18,19]. An EFO-based trait search was performed using the keyword “breast carcinoma”, and additional sub-traits were manually curated and filtered out: survival, prognosis, toxicity, therapy, density, response, radiotherapy, treat, induce, mortality, and signature. Further filtering was performed for the “European population” in either discovery or validation sample descriptions, yielding 41 studies detailing 1080 unique risk-associated variants published until 17 February 2021 (Supplementary Table S1).

### 2.2. Ancestry Analysis

We conducted Principal Component Analysis using PLINK2 (v2.00a3LM) and 1000 Genomes (1000 G) data, focusing on European ancestry [20]. VCF file processing utilised BCFTools (v1.10.2 with htslib1.10.2-3) for variant extraction and merging GTEx and 1000 G data [21,22]. As previously reported, we used the top four principal components (PCs) to improve ancestry granularity [23], classifying 711 individuals from the GTEx dataset as Europeans, with 119 having breast tissue RNA-seq data available.

### 2.3. RNA-Seq Analysis

SRA files from 119 normal breast tissue samples from female donors classified as European were converted from to FASTQ using sratoolkit (v2.10.8) [24], followed by quality control with FastQC (v0.11.9), multiQC (v1.9) [25], and cutadapt (v2.8) [26]. STAR (v2.7.5a) [27] aligned reads to the hg38 assembly [28], achieving alignment rates between 85.8% and 93.4%, resulting in 26.2 M to 62.7 M reads mapped per sample (Supplementary Figure S1).

### 2.4. Alternative Splicing Quantification

Psichomics (v1.16.0) [29] was used to quantify alternative splicing, with a minimum threshold of 10 reads per exon junction. The package maintainer provided event annotations. We only considered events with quantification for all samples and variability higher than 0 for further analysis.

### 2.5. sQTL Analysis

TensorQTL [30,31] was used for sQTL mapping in a ±1 Mb window from the 5′ boundary of the alternative event (A5SS), with the top 15 PCs as covariates. We conducted both nominal sQTL association, which utilises Pearson correlation and reports *p*-values for all tested variant–event pairs, and empirical-beta approximation, which uses a beta-approximated distribution based on permutation testing to report the best-associated variant for each event. *p*-values from both approaches were corrected for multiple testing using the Benjamani–Hochberg method (FDR), setting significant association at FDR ≤ 0.05.

sGenes from GTEx v.8 were retrieved and compared to those identified by our sQTL analysis. The comparison was not limited to genes analysed in both datasets due to the lack of information on non-significant genes in the GTEx dataset.

### 2.6. Linkage Disequilibrium Analysis

Linkage Disequilibrium (LD) analysis between sQTL and GWAS risk variants used LDlinkR (assembly GRCh37) with an r^2^ threshold ≥ 0.4, based on European super populations (including CEU, TSI, FIN, GBR, and IBS) [32,33].

### 2.7. Colocalisation Analysis

Selected loci were tested using the SuSiE implementation of COLOC [34]. Variants found both to be sQTLs in breast tissue and associated with risk for breast cancer in published GWASs were selected, and an LD matrix was retrieved using LDlinkR [33]. Variants in perfect SNPs (r^2^ = 1) were pruned, keeping the variant with the highest combined value of log *p*-values as suggested by COLOC authors, except for *BANF1* variants for which pruning was performed for variants with r^2^ ≥ 0.98 to allow computation in a very complex LD structure locus. SNP-wise priors were set at 1 × 10^−4^ for p_1_ and p_2_ and 1 × 10^−5^ for p_12_ and the colocalisation hypothesis at 0.9894755 for H_0_, 5 × 10^−3^ for H_1_ and H_2_, 2.45 × 10^−5^ for H_3_, and 5 × 10^−5^ for H_4_. True colocalisation was criteria was (1) PP(H_4_) ≥ 0.9 and (2) PP(H_4_) ≥ 3 × PP(H_3_).

### 2.8. In Silico Splicing Analysis

Variants identified as colocalised together with their proxies (r^2^ ≥ 0.8 in the European superpopulation) were annotated regarding gene location using BiomaRt (build GRCh38.p13) [35]. Variants within genes were assessed for potential impacts in splicing via in silico analysis.

NetGene2 [36] and SpliceAI [9] were used to detect changes in primary splicing elements. NetGene2 analysed a 201-nucleotide sequence from Ensembl, focusing on the variant at position 101, to record allele score changes. SpliceAI’s Online API evaluated score deltas within 50 nucleotides of the variant on hg38. The destruction of splice site predictions was only considered for variants located within 50 bps of the splice site, while predictions on creating new splice sites were considered for all variants. Encore [37], Postar3 [38], and RBP-map [39] were applied to identify changes in auxiliary splicing elements, specifically RNA-binding protein (RBP) motifs. Encore and Postar3 queries targeted genes of interest. RBP-map processed sequences of 221 nucleotides for all human RBPs to predict binding changes. HSF, composed of multiple tools, was used to predict the overall impact of changes in primary sequence on splicing [40] based on rsID input. Additionally, both SpliceAI and HSF provided a threshold to establish significant differences in the prediction of splicing impact between the two alleles of the variant.

### 2.9. eQTL Analysis

Transcript-wise eQTL analysis involved GTEx data and gene boundary information from Ensembl using BiomaRt [35], with the top 15 PCs on normalised transcript-wise counts used as covariates. Results were considered significant when FDR ≤ 0.05.

## 3. Results

**Figure 1:**
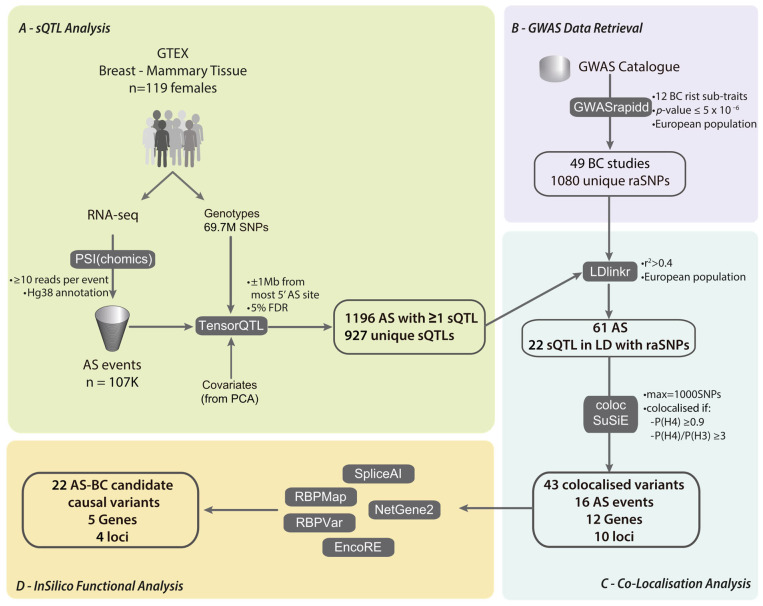
Analysis pipeline. (**A**) Quantification of alternative splicing events and sQTL mapping using data from GTEx project. (**B**) Retrieval of previously identified breast cancer risk-associated variants. (**C**) Colocalisation analysis to identify variants associated. (**D**) In silico functional analysis of putative causal variants.

To investigate the effect of alternative splicing cis-regulatory variants on breast cancer risk, we initiated our analysis with a genome-wide sQTL analysis in healthy breast tissue data, followed by a colocalisation analysis with previously reported GWAS risk-associated variants, and finishing with an in silico functional analysis (Figure 1).

### 3.1. Splicing QTL Hints at Underlying Gene Regulation

**Figure 2:**
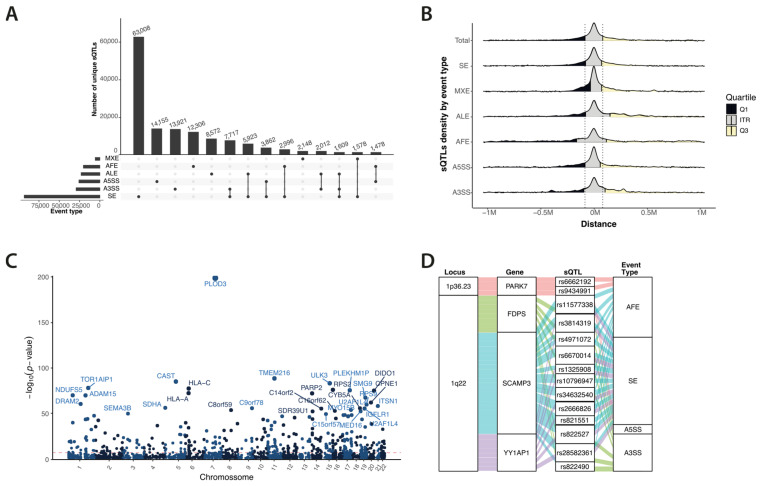
Summary of sQTL and colocalisation analysis. (**A**) Upset plot showing number of events detected as well as variants per event type. (**B**) Distance between sQTLs and mapping origin. Values are corrected for gene coding strand. (**C**) Manhattan plot of best sQTL per gene. Gene names shown when q-value ≤ 10 × 10^−50^. Red dotted line identifies significance threshold of 5 × 10^−8^. (**D**) Colocalisation results for two loci on chromosome 1. SE—skipped exon, MXE—mutually exclusive exon, ALE—alternative last exon, AFE—alternative first exon, A5SS—alternative 5′ splice site, A3SS—alternative 3′ splice site.

To reveal the effect of variants acting on alternative-splicing equilibrium in breast tissue, we performed sQTL mapping. We first quantified alternative splicing on 107,000 events across 119 samples, identifying 29,993 informative events. PSI at each event was then tested for associations with genotypes, linking over 152,000 genetic variants to changes in splicing (sQTLs) (5% FDR) in 3978 genes (sGenes). Some variants were associated with more than one event type, yielding over 241,000 unique variant–splice event pairs (Figure 2A, Supplementary Table S2). A total of 26% of the sGenes we identified overlapped with those identified in the GTEx project, while 38% were novel discoveries (see Supplementary Figure S2).

Most sQTLs were located outside annotated gene boundaries (81.86%), with only a minor fraction within gene bodies or splicing events (Supplementary Figure S3). The distribution of sQTLs relative to splicing events also varied by event type. Variants associated with alternative first exon and alternative 5′ splice site events tended to be located more upstream, whereas those associated with the alternative last exon, alternative 3′ splice site, and mutually exclusive exons showed a much narrower range of localisation. Specifically, the interquartile range (IQR) for mutually exclusive exons was 106k, compared to 170k for all events combined (Figure 2B, Supplementary Figure S4).

While the average impact of alternative alleles on splicing was modest (75% of sQTLs displayed a |slope| below 0.064 (Supplementary Figure S5)), some variants like rs12898397 located in gene *ULK3* demonstrated a substantial effect (slope = 0.5) (Supplementary Figure S6). Our permutation-based mapping highlighted the top sQTLs, identifying 926 variants associated with 1170 alternative splicing events in 726 genes, such as rs71593133 for *SDHA* (*p*-value 4.01 × 10^−57^) and rs2297616 for *PARP2* (*p*-value 6.02 × 10^−73^) (Figure 2C, Supplementary Table S3).

### 3.2. Colocalisation Implicates Splicing Modulation in Risk for BC at Ten Loci

To identify common variants that modulate breast cancer risk via their impact on splicing, we identified colocalised sQTLs with reported GWAS hit variants. We first identified 13 loci harbouring top sQTLs and breast cancer risk variants in LD (Supplementary Table S4), but one was dropped due to a lack of GWAS summary statistics.

Colocalisation analysis pinpointed 43 variants on twelve genes located in ten different loci (Supplementary Table S5), including the highly complex locus 1q22 where *FDPS*, *SCAMP3,* and *YY1AP1* share several colocalised SNPs (Figure 2D).

### 3.3. Linking Risk Variants to Mechanism at Three Loci

To pinpoint the candidate causal variant within each locus, we assessed the potential effect on splicing of the 133 variants (colocalised or in high LD with these) located within gene boundaries (Supplementary Table S6).

We identified 85 variants that showed evidence of splice site modulation. More specifically, four variants, located within *MRPL11*, *SGCE*, *FDPS,* and *YY1AP1,* were predicted to impact splicing elements (5′ and 3′ splice sites and the branching point) and were in close proximity (<50 bp) to the alternative splice element, further supporting their regulatory potential. Seventy-three variants were predicted to alter splice auxiliary elements (exonic and intronic splice enhancers and silencers), and 40 variants were found to modify RBP recognition (Supplementary Table S7).

Three variants (rs11264361, rs10247562, rs11110) showed multiple lines of evidence of altering splice elements and were analysed in more detail.

**Figure 3:**
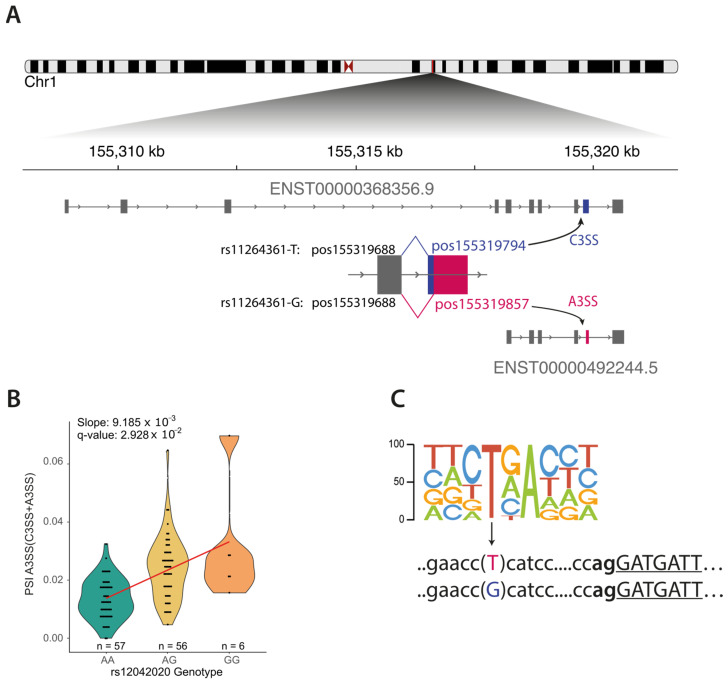
Summary of the evidence collected linking rs11264361 to the splicing regulation of FDPS. (**A**) Gene model of FDPS showing the allele-specific effect of rs11264361 on the final isoform. (**B**) sQTL for rs12042020, the best FDPS-associated variant, at the event reported in panel A. (**C**) Consensus motif for U2 snRNA, variant, and flanking sequence at the rs11264361 variant site.

The minor allele (allele G) of rs11264361, located in intron 8 of *FDPS,* was predicted to break a branching point in exon 9, changing the 3′ splice site from position 155,319,794 to position 155,319,857 (Figure 3A). This variant is in strong LD (r^2^ = 0.81) with the sQTL rs12042020, whose minor allele G we found associated with a significant increase in the ratio of alternative 3′ splice site usage for the same event (slope = 0.009, *p*-value = 1.07 × 10^−7^) (Figure 3B, Supplementary Table S4) and was also predicted to disrupt crucial splicing mechanisms, impacting the spliceosome component U2AF and altering splicing ratios (Figure 3C). eQTL analysis corroborated these results, indicating that rs11264361 was also an eQTL for specific *FDPS* isoforms (Supplementary Figure S7). rs11264361 was in LD with rs12091730 (r^2^ = 0.67), whose minor allele was associated with an increased risk for breast cancer [1]. None of these changes in alternative splicing have been reported in breast tumours [41].

Similarly, the major allele G of rs10247562, an exonic variant of *SGCE, was* predicted to decrease the exon usage where it is located (Supplementary Table S7). This variant was in LD (r^2^ = 0.98) with the sQTL rs11508502, whose major allele was found to be associated with an increase in the exon skipping (slope = −0.014, *p*-value = 5.61 × 10^−18^) (Supplementary Table S4). Furthermore, rs10247562 showed RBP binding (PIP-Seq in He-La cells), and in silico analysis predicted a change in auxiliary splicing sequences with disruption of RBP binding (Supplementary Table S7). rs10247562 was in high LD with rs17268829 (r^2^ = 0.91), whose major allele was associated with protection for BC [1,42]. Interestingly, breast tumours have shown higher inclusion of exon 12 than normal tissue (Supplementary Figure S8A) [41]. This finding aligns with our results, where lower inclusion of exon 12 was associated with protection against breast cancer.

Lastly, we found that rs11110, an exonic variant of *MRPL11* located in an auxiliary splicing sequence (enhancer or silencer), is predicted to influence the use of a 5′ splice site in *MRPL11* within the same exon, resulting in the inclusion of the variant site in the final isoform when the minor T allele is present (Supplementary Table S7). Concordantly, this variant was among the top sQTLs (Supplementary Table S3), with the T allele associated with increased usage of the same 5′ splice site in *MRPL11* (slope = −0.088, *p*-value = 1.42 × 10^−25^). Additionally, CLIP data show the binding of various RBPs at the variant site in multiple cell lines (Hek293, K562, HepG2), with the major allele C showing higher affinity for these RBPs (Supplementary Table S7). This variant was in LD with rs1134495 and rs7570 (r^2^ = 0.41 and 0.47, respectively), whose minor alleles have been reported to be associated with risk for breast cancer [1,42]. These findings are discordant from previously reported preferential usage of the longer exon 1 in tumour tissue (Supplementary Figure S8B) [41].

## 4. Discussion

Given the excess of intronic and intergenic breast cancer risk-associated variants identified in GWASs [4], previous studies have intended to establish a link between risk-associated variants and alternative splicing. However, they were limited by considering alternative splicing events in tumour tissue rather than normal [16,17], focusing on a single variant [15] or a small set [17], and not establishing causality by lack of colocalisation analysis [16,17].

This study provides the first compelling evidence that genetic variants contribute to breast cancer risk by impacting on alternative splicing in the normal breast. We identified ten loci where the top sQTL variant colocalises with previously reported breast cancer risk variants, suggesting that splicing modulation may be the mechanism driving risk at these loci. Additionally, we identified rs11264361, rs10247562, and rs11110 as candidate causal variants that affect the splicing of the genes *FDPS*, *SGCE*, and *MRPL11*, respectively, in this context.

Our analysis began with an sQTL analysis identifying variants associated with splicing modulation for 3978 genes. Due to the prevalence of breast cancer GWASs in European ancestry populations [43], our analysis focused on individuals of this same ancestry. Furthermore, we exclusively analysed RNA-seq data from healthy female breast tissue as hormones, like oestrogen and progesterone, may alter gene expression regulation between genders [44] and because of the higher incidence of breast cancer in females [45].

We revealed a significant portion of unique sGenes in our study compared to published sGenes for GTEx [46], underscoring the importance of ancestry and gender in gene expression analysis. The comparison was not limited to genes analysed in both datasets, and our use of annotation-based tools for detecting alternative splicing (different from those used by GTEx) may have missed certain events, such as cryptic splice sites. This highlights the need for more comprehensive methods, like full-length mRNA sequencing, when performing such comparisons [28,47]. In addition, the presence of variants in trans, affecting RBPs expression, may also be an important factor to take into account in a further study.

Our findings show that the majority of sQTLs are located within 170 Kb of the associated splicing event and outside gene boundaries and beyond typically considered ranges in previous studies (5 Kb from the gene boundary or 100 Kb from the tested event) [46,48], suggesting the existence of distant regulatory elements affecting splicing, such as transcript-specific promoters/enhancers, and mechanisms related to chromatin accessibility and RNA polymerase II, as previously reported [28,48,49,50,51]. However, we cannot exclude the fact that the sQTLs we identified may be capturing the effect of high-LD regulatory variants within gene boundaries. The distribution of sQTLs by event type suggests differing underlying mechanisms. Mutually exclusive exon events appear to be more sensitive to proximal variants, while alternative first-exon events are more influenced by distant regulatory elements.

Our colocalisation analysis identified ten loci where variants cis-regulating splicing are candidate causal variants for breast cancer risk. This indicates that while splicing regulation may not be the most common mechanism driving risk, it is significant and should not be overlooked. Our results may even underestimate the impact of this mechanism because the colocalisation analysis was limited to loci with available GWAS summary statistics, excluding other loci where sQTLs were identified, such as *NR1H3*. In addition, extending this study to include other populations may yield broader insights into susceptibility to breast cancer-specific subtypes.

Some of the genes in the colocalised loci, including the ones functionally characterised, have been previously linked to tumour development. For instance, FDPS is involved in cholesterol biosynthesis and prenylation, processes vital for cell functions often exploited by cancer cells [52,53]. SGCE’s role in cell structure and signalling suggests its involvement in cancer progression [54,55], while MRPL11’s involvement in mitochondrial metabolism points to its potential role in cancer biology [56,57]. These changes are often linked to oncogenic processes, highlighting the potential for targeted therapies using transcript-specific RNAi or serving as neoepitopes, a target for immunotherapy.

The complexity of splicing and its context-dependent nature underscores the need for more targeted approaches in understanding and modelling this mechanism. This requires advanced techniques like eCLIP experiments on relevant tissue, such as breast, to improve our understanding of RBPs’ roles in splicing and breast cancer risk. Hence, the addition of experimental validation in future studies will support the importance of the variants identified herein and their inclusion in risk assessment tools.

## 5. Conclusions

Our study sheds new light on how cis-regulatory variants involved in alternative splicing contribute to the increased risk of breast cancer. By identifying twelve genes within ten loci where top sQTL variants colocalise with previously reported breast cancer risk variants, we implicate splicing modulation as a key mechanism driving risk at these loci. Nevertheless, limitations in data availability prevented comprehensive colocalisation analysis for some genes associated with risk for breast cancer and for which sQTL were identified. In the future, analysis using updated GWAS, RNA-seq, and genotyping data, including data from different populations, and experimental validation will provide definite support for our findings.

Overall, our study emphasises the importance of exploring splicing regulation in the context of cancer risk, mainly through a tissue- and gender-specific lens.

## Data Availability

Data generated in this study are provided as Supplemental Data, and the code can be made available upon request to the corresponding author. GWAS summary statistics were obtained from the GWAS Catalog website via FTP download http://ftp.ebi.ac.uk/pub/databases/gwas/summary_statistics/GCST004001-GCST005000/GCST004988/ (accessed on 28 January 2021) and http://ftp.ebi.ac.uk/pub/databases/gwas/summary_statistics/GCST007001-GCST008000/GCST007236/ (accessed on 27 January 2021). SNP data were obtained from the Ensembl database (versions 92 and 75) and are available at www.ensembl.org (accessed on 30 June 2024). Gene and transcript expression and eQTL data for breast tissue from the GTEx Project (v7) were retrieved from the GTEx Portal at www.gtexportal.org (accessed on 30 June 2024). Other detailed results are available in Supplementary Data.

## Supplementary Materials

The following supporting information can be downloaded at https://www.mdpi.com/article/10.3390/cancers16173020/s1: Supplemental Data include seven tables and eight figures. Supplementary Table S1: List of GWAS retrieved from the GWAS Catalog. study_id—unique identifier assigned by GWAS Catalog, pubmed_id—PubMed identification number, publication_date—original publication date, publication title—Title of paper, author_fullname—first author full name, author_orcid—first author orcid. Supplementary Table S2: List of significant sQTLs. phenotype_id—identification of alternative splicing events, variant_id—position-based identification of associated variant, tss_distance—distance between 5′ most splice site to associated variant, af—allele frequency, ma_samples—number of samples with the minor allele, ma_count—total count of minor alleles across all samples, pval_nominal—The nominal p-value of the association between the variant and the phenotype, slope—The beta (slope) of the linear regression, slope_se—The standard error of the beta, FDR_pval—FDR corrected p-value of association, log10FDR—negative logarithmic transformation of the FDR corrected p-value. Supplementary Table S3: Significant sQTLs using a beta-approximated distribution based on permutation testing. Supplementary Table S4: Variants of interest per loci and event. Supplementary Table S5: Colocalisation of sQTL and GWAS from summary statistics. Supplementary Table S6: Variants Colocalized or in high LD of interest. Supplementary Table S7: In silico Splice Analysis of variants of interest. Supplementary Figure S1: RNA-seq alignment rates of normal breast samples from the GTEx project. Supplementary Figure S2: sGenes overlapped with GTEx breast tissue analysis.Supplementary Figure S3: Relative position of each alternative splicing associated variant relative to the gene and the alternative splice event, corrected for strand. Supplementary Figure S4: Distribution of distances from alternative splice 5’ most edge to associated variant. Supplementary Figure S5: Distribution of the absolute slope value across negative logarithm of the p-value. Supplementary Figure S6: Example of an extreme effect, where alternative allele rs12898397-C content is associated with a decrease in PSI levels of 0.5125, depleting the reference splice pattern in favour of the alternative.Supplementary Figure S7: Transcript-wise QTL of FDPS at rs11264361. *p*-value and slope are provided for significant QTLs. Supplementary Figure S8: Changes in splicing between breast tumour and normal-matched tissue for the alternative splice events of interest.
