# Supplementary material for: Colocalised Genetic Associations Reveal Alternative Splicing Variants as Candidate Causal Links for Breast Cancer Risk in 10 Loci
Source: Cancers (Basel). 2024 Aug 29;16(17):3020. doi: 10.3390/cancers16173020 (PMC11394352; doi:10.3390/cancers16173020)
Supplement: Supplementary file 1 [file cancers-16-03020-s001.zip › Besouro-Duarte_RpR_SuppMaterial/Besouro-Duarte2024_SupplementaryMaterials.pdf]

Supplementary Materials to:

COLOCALISED GENETIC ASSOCIATIONS REVEAL ALTERNATIVE SPLICING VARIANTS AS CAUSAL LINKS FOR BREAST CANCER RISK IN 10 LOCI

André Besouro-Duarte [a], Beatriz Carrasqueiro [b], Sofia Sousa [b], Joana M. Xavier [a,c], Ana-Teresa Maia [a,b,c]¶

a. CINTESIS@RISE, Universidade do Algarve, Faro, Portugal
b. Faculty of Medicine and Biomedical Sciences, Gambelas Campus, Universidade do Algarve, Faro, Portugal
c. Centro de Ciências do Mar (CCMAR), Universidade do Algarve, Faro, Portugal

¶ - Corresponding Author: atmaia@ualg.pt

Supplementary Table 1: List of GWAS retrieved from the GWAS Catalog. study_id – unique identifier assigned by GWAS Catalog, pubmed_id – PubMed identification number, publication_date – original publication date, publication title - Title of paper, author_fullname – first author full name, author_orcid – first author orcid.

Supplementary Figure 1: RNA-seq alignment rates of normal breast samples from the GTEx project. The x-axis represents the number of uniquely aligned reads per sample in millions, and the y-axis represents the percentage of reads successfully aligned.

Supplementary Table 2: List of significant sQTLs. phenotype_id – identification of alternative splicing events, variant_id – position-based identification of associated variant, tss_distance – distance between 5' most splice site to associated variant, af – allele frequency, ma_samples – number of samples with the minor allele, ma_count – total count of minor alleles across all samples, pval_nominal - The nominal p-value of the association between the variant and the phenotype, slope - The beta (slope) of the linear regression, slope_se - The

standard error of the beta, FDR_pval – FDR corrected p-value of association, log10FDR – negative logarithmic transformation of the FDR corrected p-value.

Supplementary Figure 2: sGenes overlapped with GTEx breast tissue analysis.

Supplementary Figure 3: Relative position of each alternative splicing associated variant relative to the gene and the alternative splice event, corrected for strand. AFE – Alternative First Exon, A5SS – Alternative 5' Splice site, SE – Skipped Exon, MXE – Mutually Exclusive Exon, A3SS – Alternative 3' Splice Site, ALE – Alternative Last Exon, out_gene_5 – variant upstream of the annotated gene, in_gene_5 – variant within annotated gene, between transcription start site and alternative splice event, in_event – variant within annotated alternative splice event, in_gene_3 – variant within annotated gene, between alternative splice event and gene end, out_gene_3 – variant downstream of the annotated gene.

Supplementary Figure 4: Distribution of distances from alternative splice 5' most edge to associated variant. A significant difference between groups was observed (Kruskal-Wallis chi-squared = 458.4, df = 6, p-value < $2.2.e^{-16}$).

Supplementary Figure 5: Distribution of the absolute slope value across negative logarithm of the p-value. Most sQTLs display a modest effect size (percentile 90 is 0.1).

Supplementary Figure 6: Example of an extreme effect, where alternative allele rs12898397-C content is associated with a decrease in PSI levels of 0.5125, depleting the reference splice pattern in favour of the alternative.

Supplementary Table 3: Significant sQTLs using a beta-approximated distribution based on permutation testing. Only the lowest p-value per alternative splice event is reported. Phenotype_id – alternative splice event as identified by psichomics, num_var – number of tested variants, beta_shape1 and beta_shape2 – beta distribution parameters, true_df – true distribution, pval_true_distribution – beta approximated p-value, variant_id – position based variant identification, tss_distance – distance from 5' most edge of the splice site to the variant position, ma_samples – samples with minor alleles, ma_count – total number of

minor alleles across all samples, af – allele frequency, pval_nominal – p-value of association of nominal mapping, slope – effect size of the linear regression, slope_se – standard error of the effect size, pval_perm – p-value obtains from the permutation testing, pval_beta – beta-approximated p-values.

Supplementary Table 4: Variants of interest per loci and event. sQTL chr – chromosome where sQTL is present, locus gene – locus where sQTL is in LD with GWAS hit-SNP, Phenotype_id – alternative splice event as identified by psichomics, slope – effect size of the linear regression, slope_se – standard error of the effect size, pval FDR – FDR corrected p-value of association of the sQTL, pval FDR (exp) – Exponent value of the FDR-correct p-value of association, sQTL id – variant RsId, sQTL pos – sQTL position, sQTL ref all – sQTL reference allele, sQTL alt all – sQTL alternative allele, GWAS hit SNP – RsId of the GWAS hit-SNP, LD (r2) – Linkage Disequilibrium (r2) between sQTL and GWAS hit-SNP, GWAS_pos – GWAS hit-SNP position, GWAS pvalue - GWAS hit-SNP p-value of association, GWAS pvalue (exp) - Exponent value of the GWAS p-value of association, GWAS risk allele – GWAS hit-SNP risk-associated allele, association id – GWAS Catalog unique identifier, complete pvalue set – availability of GWAS summary statistics

Supplementary Table 5: Colocalisation of sQTL and GWAS from summary statistics. Event – Alternative splice event; nsnps – number of SNPs tested; original_sqtl – Best associated sQTL per permutation QTL mapping; original_gwas – GWAS reported hit-SNP; Colocalized_SNP – variant colocalised; H3 – Posterior probability of the variant being associated with both traits but with distinct causal variant; H4 – Posterior probability of the variant being associated with both traits with shared causal variant.

Supplementary Table 6: Variants Colocalized or in high LD of interest. Variant_id – RsId of the variant of interest; Coord – Coordinates of the variant of interest; Alleles – Reference and alternative alleles; MAF – Minor allele frequency; colocalised – RsId of colocalised variant; R2 – Linkage disequilibrium between colocalised id and variant of interest; Correlated_Alleles – correlated alleles between colocalised variant and variant of interest; event – Annotated alternative splicing event; gene_start – Annotated gene start; gene_end - Annotated gene end; locus – chromosomal locus; event_start – 5' most edge of the annotated event; event_end – 3' most edge of the annotated event.

Supplementary Figure 7: Transcript-wise QTL of FDPS at rs11264361. P-value and slope are provided for significant QTLs.

Supplementary Table 7: In silico Splice Analysis of variants of interest. variant_id – RsId of variant of interest; Coord – genomic coordinates of variant of interest; Alleles – Reference and alternative alleles of variant of interest; gene-gene in which this variant is present; rel_pos – relative position to the annotated event (in event or gene); min_dist_to_event_splice_site – distance to closest splice site; Splice Core Elements – Prediction of change in splicing core elements (as predicted by NetGene, SpliceAI or HSF); Splice Auxiliary Elements – Prediction of changes in splicing auxiliary elements (as predicted by HSF and RBPmap); RBP binding – in vitro evidence of RBP occupation or differential binding; NetGene – summarised results of NetGene; SpliceAI– summarised results of SpliceAI; HSFcore – summarised results of HSF (splice core elements); HSFaux - – summarised results of HSF (splice auxiliary elements); RBPmap – summarised results ofRBPmap; Postar3 – summarised results of Postar3.

Supplementary Figure 8: Changes in splicing between breast tumour and normal-matched tissue for the alternative splice events of interest. A) Changes in PSI for exon 12 (12:94598004-94597930) of *SGCE* gene. B) Alternative splice of 5' splice site of *MRPL11* gene where the first exon length is reduced replacing the longer exon ENSE00002177786 (11:66,438,848-66,438,632) for the shorter ENSE00001801232 (11: 66,438,848-66,438,710).
