# Supplementary material for: Colocalised Genetic Associations Reveal Alternative Splicing Variants as Candidate Causal Links for Breast Cancer Risk in 10 Loci
Source: Cancers (Basel). 2024 Aug 29;16(17):3020. doi: 10.3390/cancers16173020 (PMC11394352; doi:10.3390/cancers16173020)
Supplement: Supplementary file 1 [file cancers-16-03020-s001.zip › Besouro-Duarte_RpR_SuppMaterial/Supplementary Figures/Supplementary Figure 4.pdf]

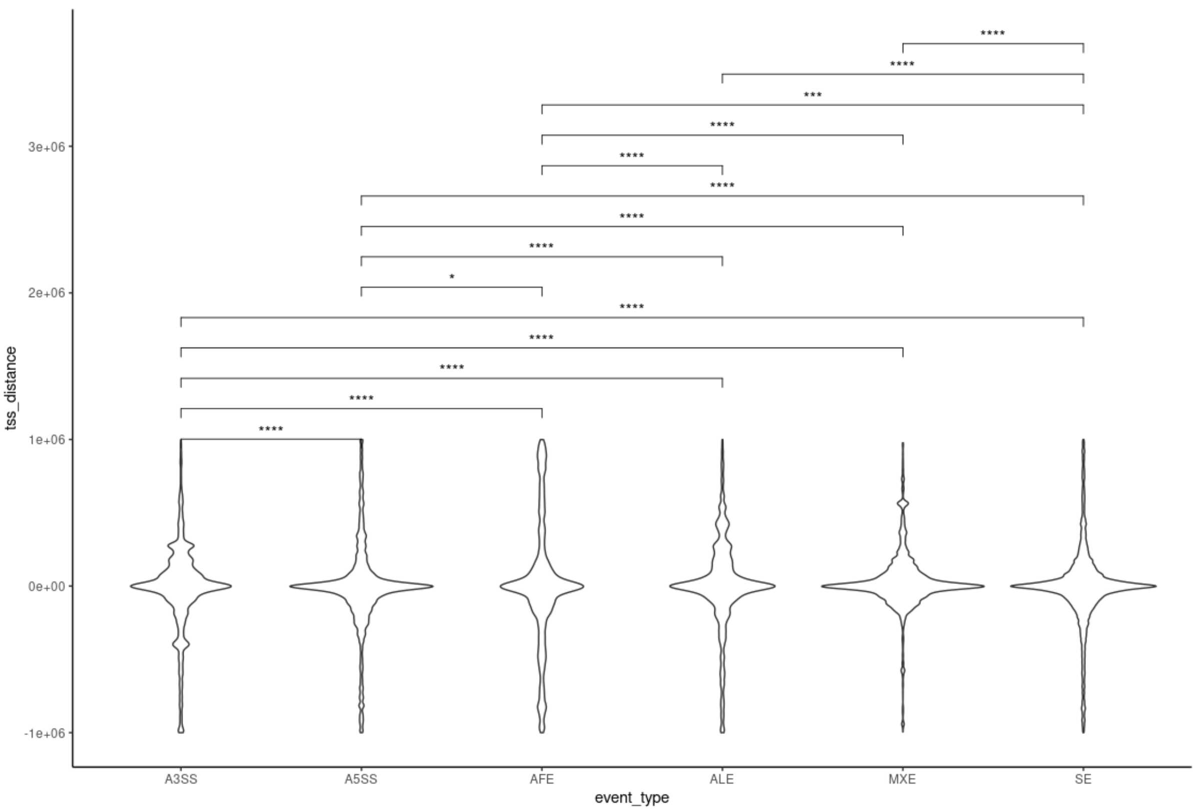

group1	group2	n1	n2	statistic	p	p.adj	p.adj.signif
A3SS	A5SS	36717	27346	530326315.5	2.4e-34	2.88e-33	****
A3SS	AFE	36717	22244	421279345.5	1.15e-10	6.9e-10	****
A3SS	ALE	36717	21770	388267094	7.73e-9	3.87e-8	****
A3SS	MXE	36717	7081	125124792	5.7e-7	2.28e-6	****
A3SS	SE	36717	118555	2242289382	1.83e-18	1.28e-17	****
A5SS	AFE	27346	22244	300466145.5	0.02	0.041	*
A5SS	ALE	27346	21770	273346047	1.04999999999999e-54	1.57e-53	****
A5SS	MXE	27346	7081	85505409.5	4.92999999999995e-52	6.9e-51	****
A5SS	SE	27346	118555	1562332258.5	9.2000000000001e-21	7.36e-20	****
AFE	ALE	22244	21770	229102425.5	1.49e-22	1.3400000000002e-21	****
AFE	MXE	22244	7081	72142568.5	1.61e-26	1.61e-25	****
AFE	SE	22244	118555	1296654501	8.16e-5	2.45e-4	***
ALE	MXE	21770	7081	76078485.5	0.101	0.101	ns
ALE	SE	21770	118555	1361179059	6.59e-38	8.57e-37	****
MXE	SE	7081	118555	452551994.5	1.83e-28	2.01000000000003e-27	****
