# Supplementary figures and images for: Colocalised Genetic Associations Reveal Alternative Splicing Variants as Candidate Causal Links for Breast Cancer Risk in 10 Loci
Source: Cancers (Basel). 2024 Aug 29;16(17):3020. doi: 10.3390/cancers16173020 (PMC11394352; doi:10.3390/cancers16173020)

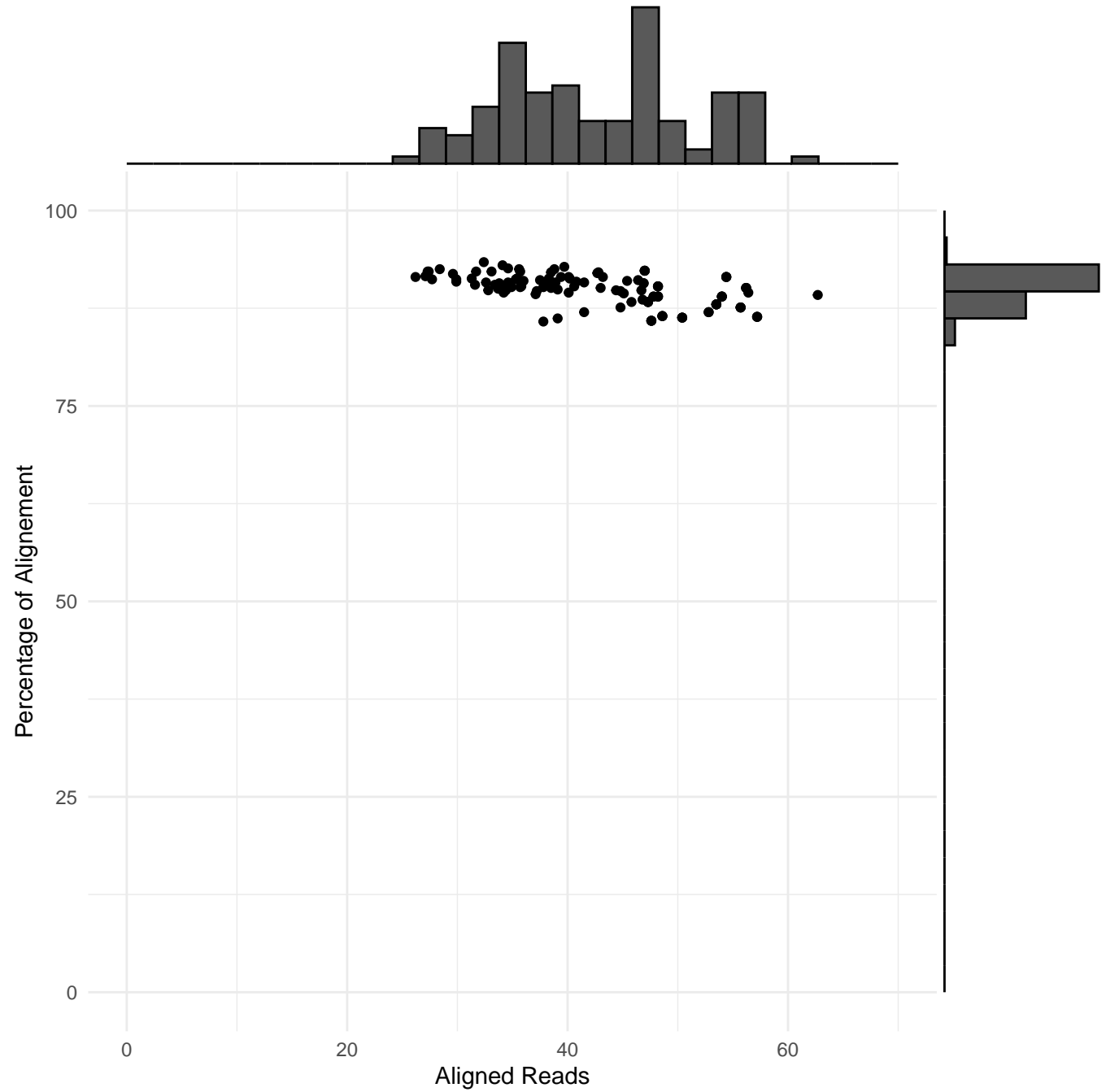

Supplement: Supplementary file 1 [file cancers-16-03020-s001.zip › Besouro-Duarte_RpR_SuppMaterial/Supplementary Figures/Supplementary Figure 1.pdf]

gtex

own

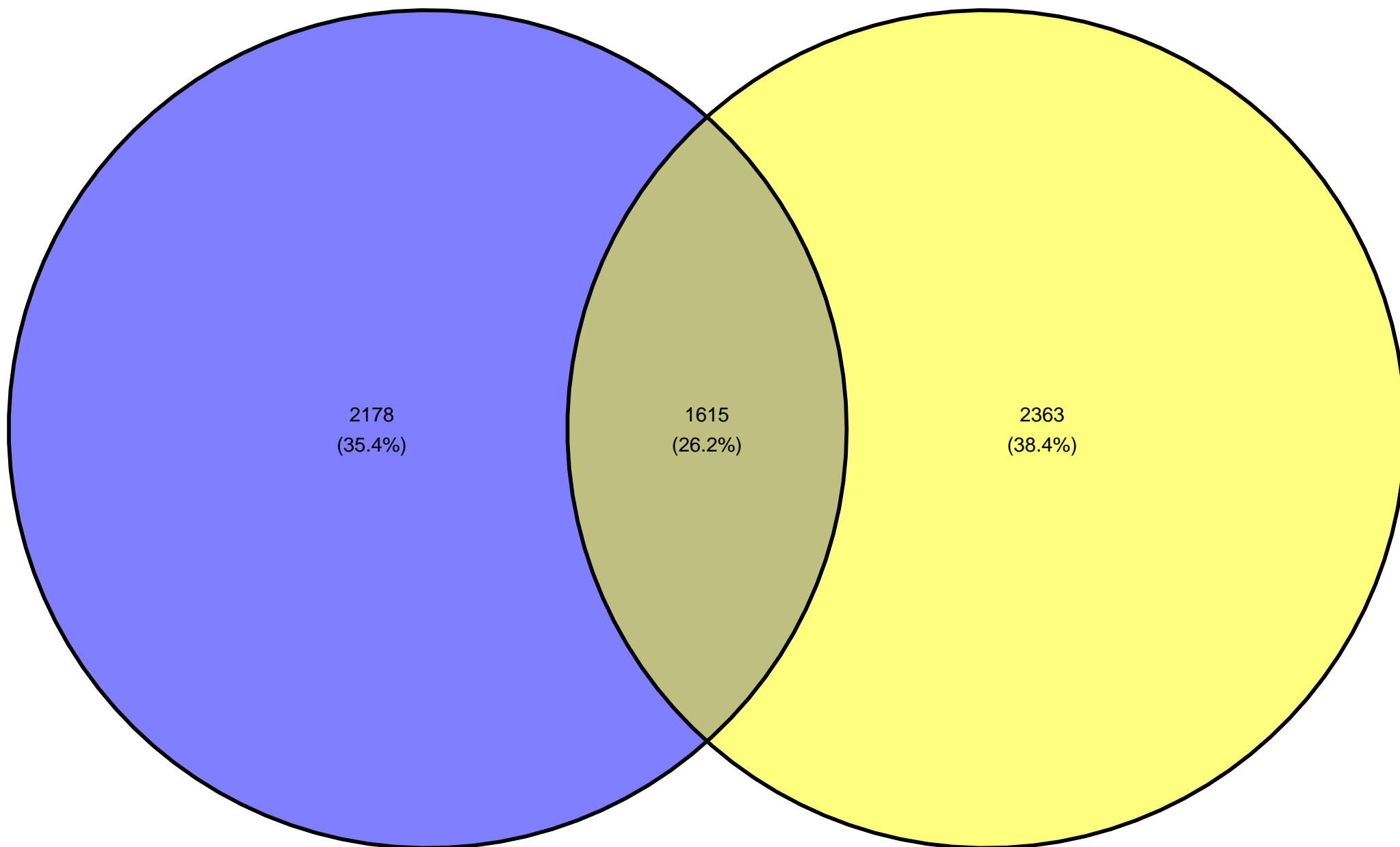

Supplement: Supplementary file 1 [file cancers-16-03020-s001.zip › Besouro-Duarte_RpR_SuppMaterial/Supplementary Figures/Supplementary Figure 2.pdf]

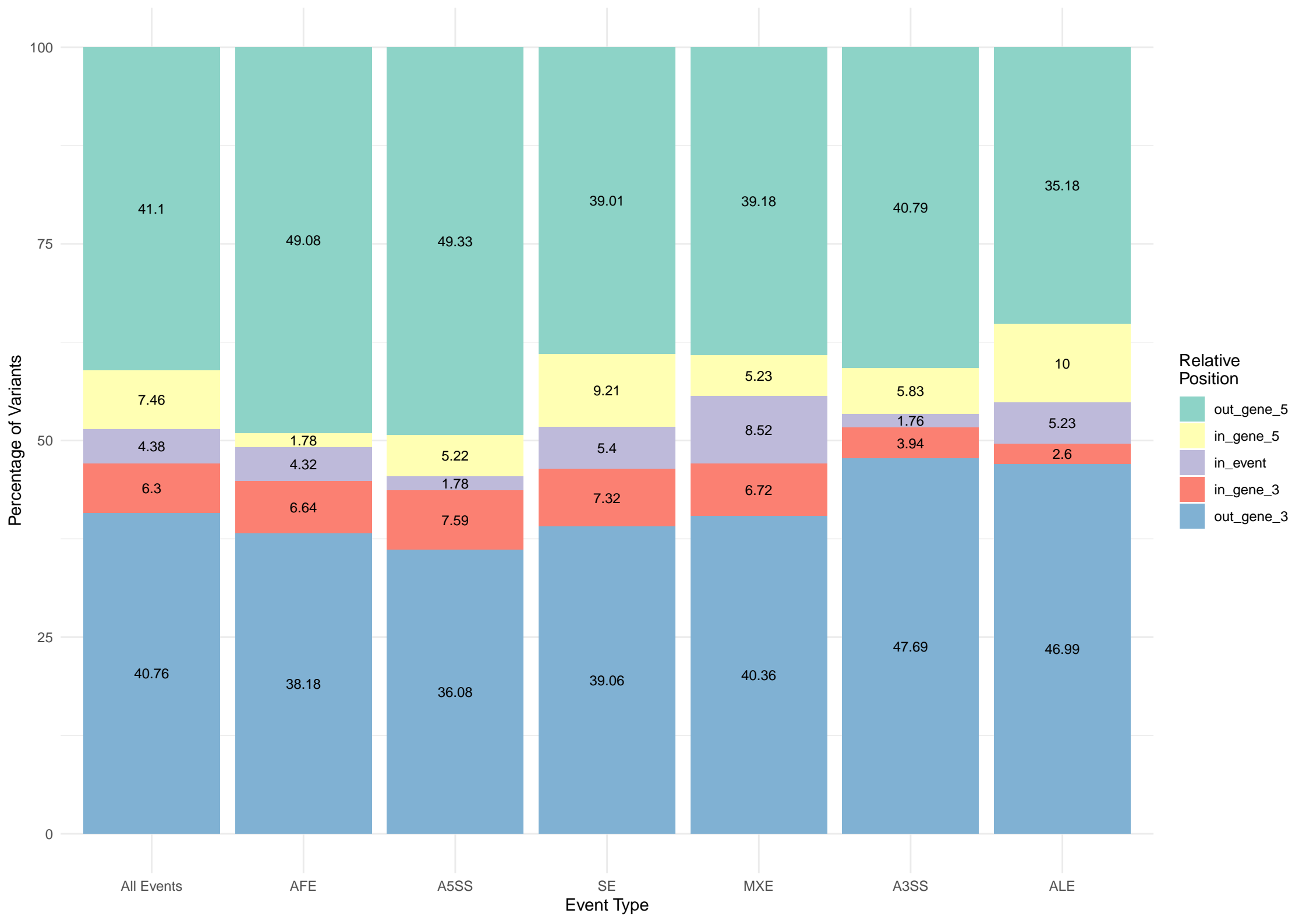

Supplement: Supplementary file 1 [file cancers-16-03020-s001.zip › Besouro-Duarte_RpR_SuppMaterial/Supplementary Figures/Supplementary Figure 3.pdf]

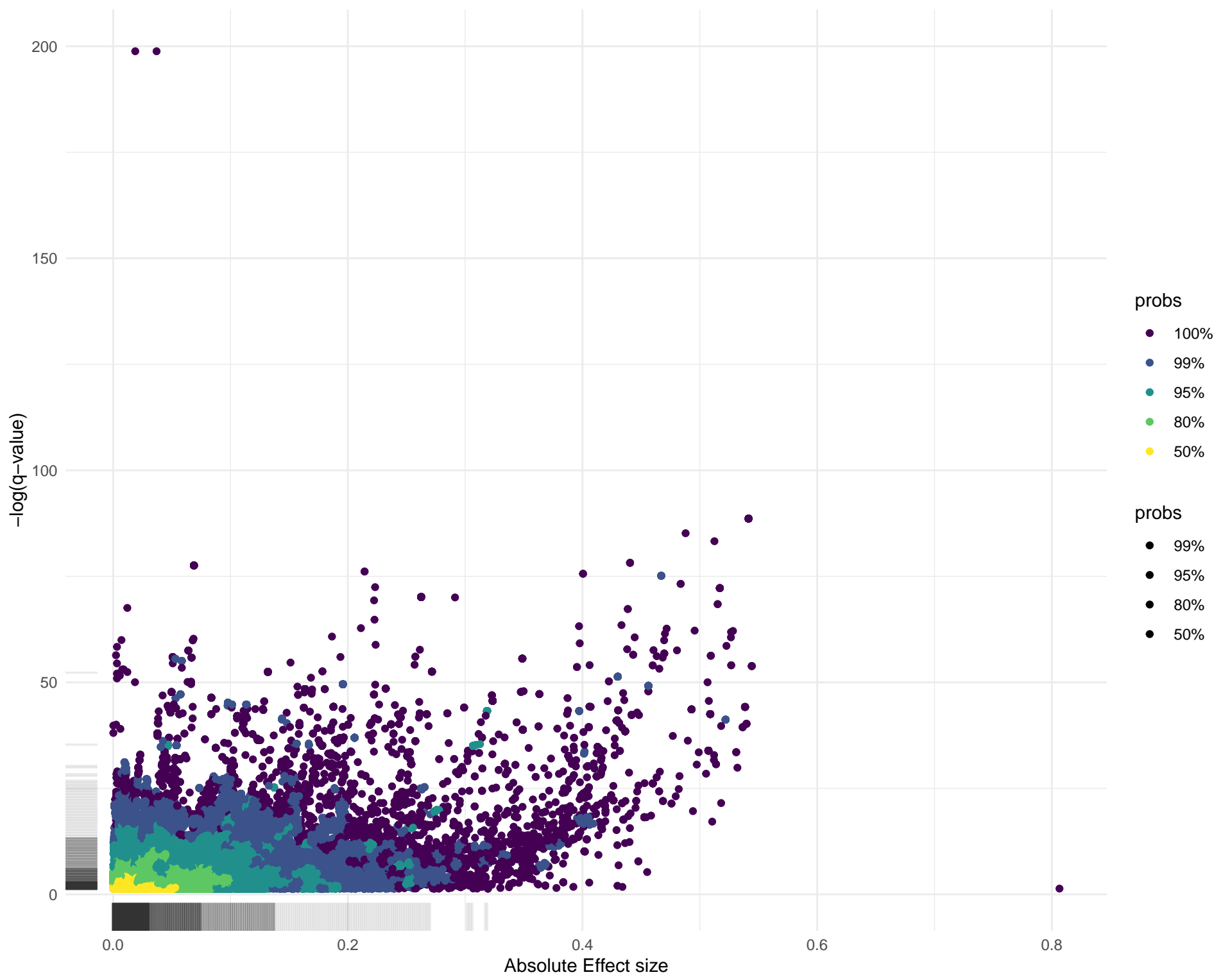

|   | mean  | sd    | IQR   | 0% | 25%  | 50%   | 75%   | 90% | 99%   |
|---|-------|-------|-------|----|------|-------|-------|-----|-------|
| 1 | 0.046 | 0.054 | 0.054 | 0  | 0.01 | 0.029 | 0.063 | 0.1 | 0.278 |

Supplement: Supplementary file 1 [file cancers-16-03020-s001.zip › Besouro-Duarte_RpR_SuppMaterial/Supplementary Figures/Supplementary Figure 5.pdf]

A5SS\_15\_-\_74837757\_74837751\_74837435\_ULK3

rs12898397

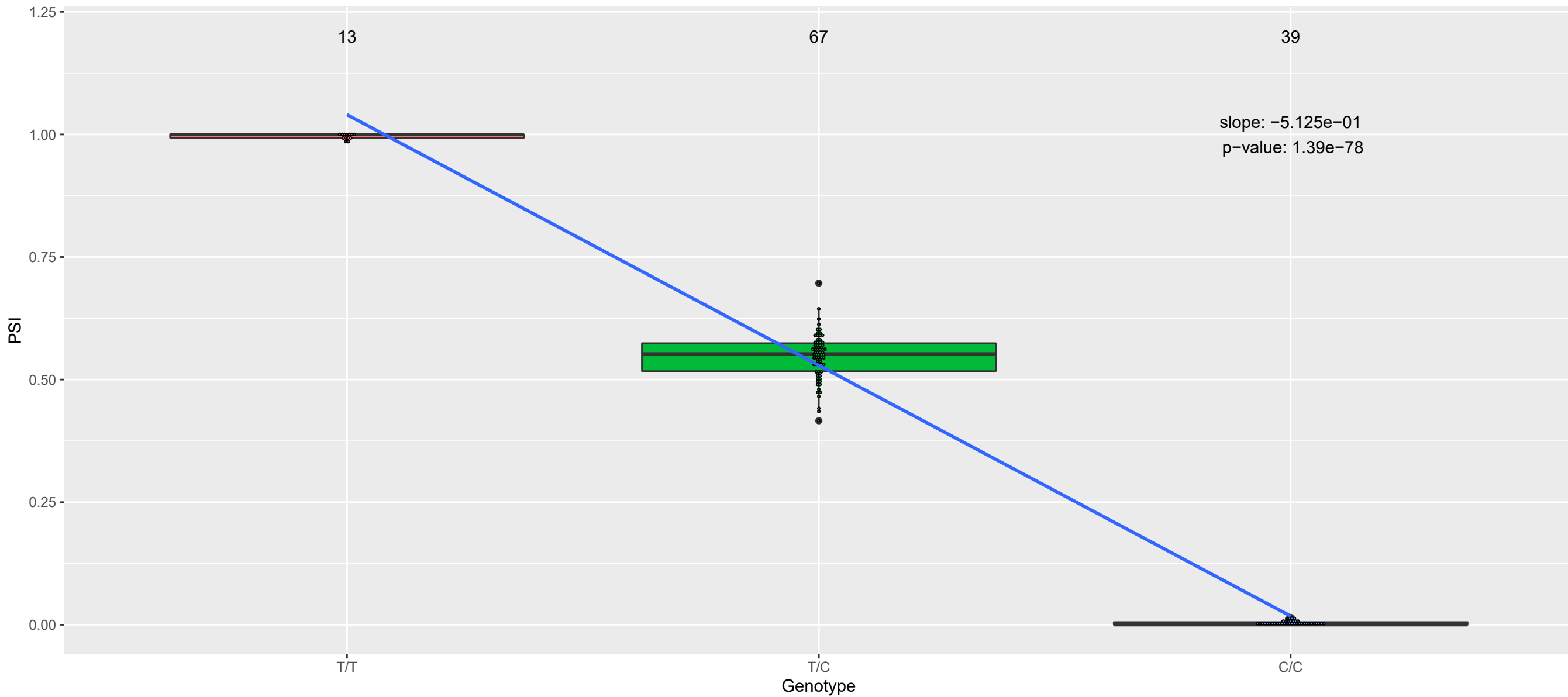

Supplement: Supplementary file 1 [file cancers-16-03020-s001.zip › Besouro-Duarte_RpR_SuppMaterial/Supplementary Figures/Supplementary Figure 6.pdf]

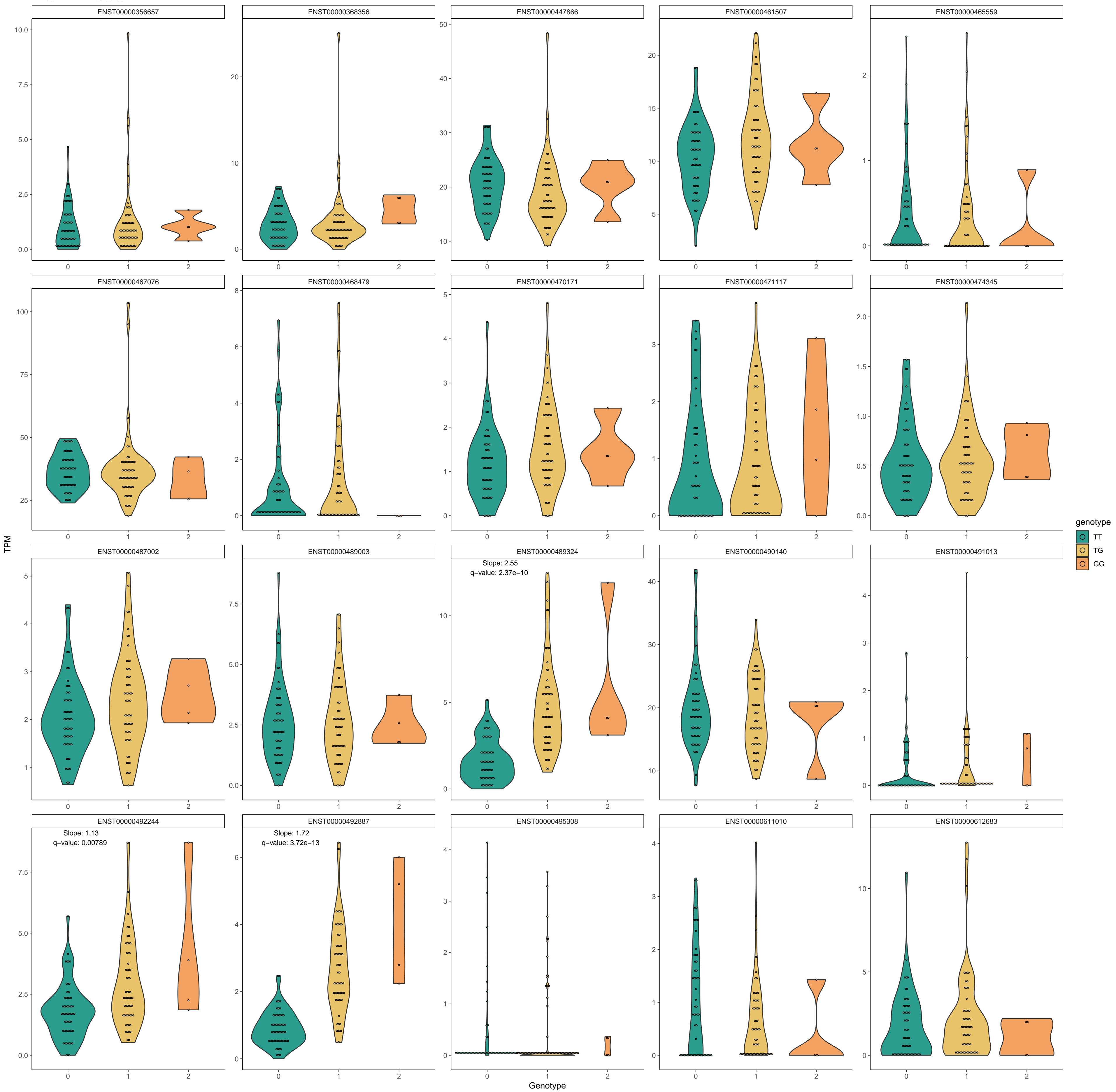

Supplement: Supplementary file 1 [file cancers-16-03020-s001.zip › Besouro-Duarte_RpR_SuppMaterial/Supplementary Figures/Supplementary Figure 7.pdf]

**A**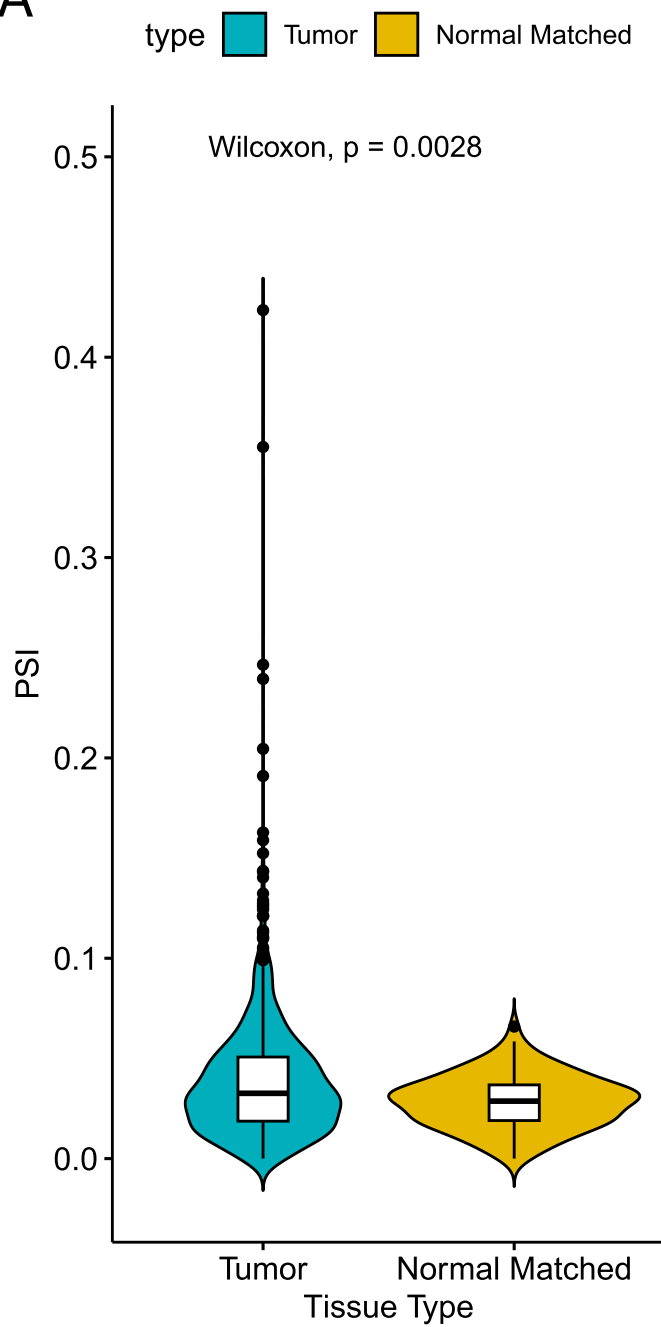**B**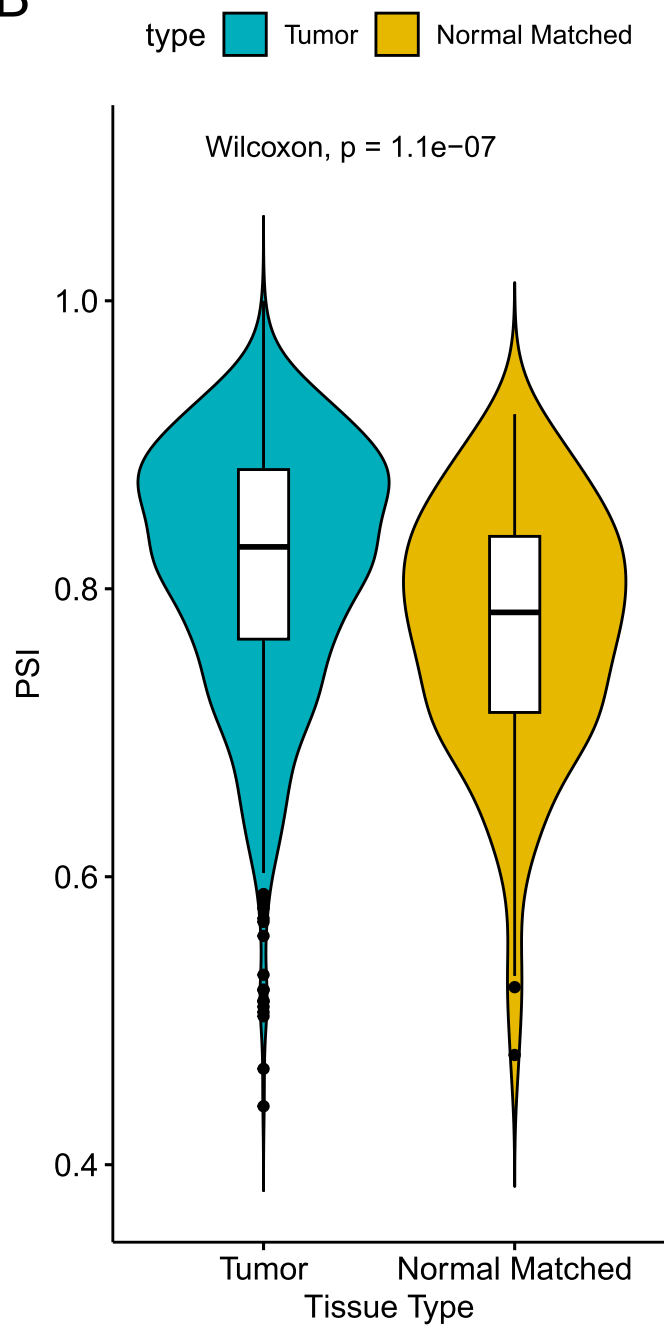

Supplement: Supplementary file 1 [file cancers-16-03020-s001.zip › Besouro-Duarte_RpR_SuppMaterial/Supplementary Figures/Supplementary Figure 8.pdf]
